# Rhamnogalacturonan I with β-(1,4)-Galactan Side Chains as an Ever-Present Component of Tertiary Cell Wall of Plant Fibers

**DOI:** 10.3390/ijms242417253

**Published:** 2023-12-08

**Authors:** Tatyana Chernova, Polina Mikshina, Anna Petrova, Nadezhda Ibragimova, Marina Ageeva, Tatyana Gorshkova

**Affiliations:** 1 Laboratory of Plant Cell Growth Mechanisms, Kazan Institute of Biochemistry and Biophysics, FRC Kazan Scientific Center of RAS, Lobachevsky Str., 2/31, 420111 Kazan, Russia; anna.petrova@kibb.knc.ru; 2 Laboratory of Plant Glycobiology, Kazan Institute of Biochemistry and Biophysics, FRC Kazan Scientific Center of RAS, Lobachevsky Str., 2/31, 420111 Kazan, Russia; p.mikshina@gmail.com (P.M.); nibra@yandex.ru (N.I.); 3 Microscopy Cabinet, Kazan Institute of Biochemistry and Biophysics, FRC Kazan Scientific Center of RAS, Lobachevsky Str., 2/31, 420111 Kazan, Russia; mageeva58@mail.ru

**Keywords:** cell wall, dynamic light scattering, immunochemistry, NMR, plant fibers, polysaccharides, rhamnogalacturonan I

## Abstract

The cellulose-enriched tertiary cell walls present in many plant fibers have specific composition, architecture, machinery of formation, and function. To better understand the mechanisms underlying their mode of action and to reveal the peculiarities of fibers from different plant species, it is necessary to more deeply characterize the major components. Next to overwhelming cellulose, rhamnogalacturonan I (RG-I) is considered to be the key polymer of the tertiary cell wall; however, it has been isolated and biochemically characterized in very few plant species. Here, we add RG-I to the list from the phloem fibers of the *Phaseolus vulgaris* stem that was isolated and analyzed by nuclear magnetic resonance (NMR), dynamic light scattering, and immunolabeling, both within tissue and as an isolated polymer. Additionally, fibers with tertiary cell walls from nine species of dicotyledonous plants from the orders Malphigiales, Fabales, and Rosales were labeled with RG-I-related antibodies to check the presence of the polymer and compare the in situ presentation of its backbone and side chains. The obtained results confirm that RG-I is an obligatory polymer of the tertiary cell wall. However, there are differences in the structure of this polymer from various plant sources, and these peculiarities may be taxonomically related.

## 1. Introduction

Plant cell specialization in the course of tissues development is often based on the deposition of the cell wall with specific properties. An impressive example is the formation of the thickened tertiary cell wall in fibers of many plant species, including the important fiber crops, like flax, hemp, ramie, etc. The tertiary cell wall is a highly cellulosic fiber-specific cell wall layer that is deposited after the secondary cell wall and is quite distinct from it in composition, architecture, function, and the whole machinery of formation [1,2]. Fibers with tertiary cell wall, or G-fibers, are widespread in the plant kingdom; they are part of various tissues and organs and are formed from the primary and secondary meristems [1,3,4]. G-fibers perform various functions, such as returning the bent shoot to a vertical position, holding the ripe fruit, deepening geophytes in soil, fixing climbing plants to a support by means of tendrils and stems, or simply maintaining a prosenchymal stem in an upright position [1,4]. Tension wood fibers from *Populus* spp., as well as fibers from agricultural crops, such as flax and hemp, are commonly used as models to study the organization and function of the tertiary cell wall, which has also been called the G-layer of the secondary cell wall [5,6,7,8,9].

The key components of tertiary cell walls are cellulose, which highly overwhelms all other polymers, reaching 80% and more of the cell wall dry weight, and rhamnogalacturonan I (RG-I) with long side chains made of β-(1,4)-galactans [2]. Within the tertiary cell wall, RG-I is considered the causative agent of tension emerging in laterally interacting cellulose microfibrils; this tension provides contractile properties to fibers and serves as the basis of their function [1].

The study of RG-I from tertiary cell walls, its structure, properties, and metabolism is instrumented by the possibility of obtaining it before and after incorporation into the cell wall. In newly synthesized RG-I from phloem fibers of flax stem—the best studied example of tertiary cell wall deposition, at least in non-woody plants—the content of galactose is so high that the polymer was first called “galactan” [10]. Later, the presence of the RG-I backbone and the linkage between rhamnose and galactose were confirmed [11,12]. Due to the intensive biosynthesis and peculiar secretion process in the course of tertiary cell wall formation, RG-I accumulates in Golgi vesicles [1] and, together with the secreted but not yet fixed within cell wall molecules, moves into the buffer-soluble fraction and elutes in the high-molecular-weight peak [10]. Within the tertiary cell wall, this polymer becomes entrapped by laterally interacting cellulose microfibrils, causing tension in the latter [1]. The entrapped RG-I can be extracted only after cellulose dissolution [11]. The intensive post-synthetic modification of RG-I by tissue-specific galactosidase leads to the shortening of side chains (coupled with the reduction in galactose content), transformation of the cell wall structure [1,13], and significant maturation of the mechanical properties of the cell wall [14]. Altogether, the version of RG-I formed in fibers during tertiary cell wall deposition is a rare example of a tissue- and stage-specific cell wall polysaccharide with confirmed importance for tissue physiological function and the possibility to isolate a particular polymer before and after incorporation into the cell wall, making it important both for the studies of fiber development and general cell wall research.

Pectic polymers in tertiary cell walls are revealed by various microscopic techniques, including immunohistochemistry, in many plant species [1,7,10,15,16,17,18]. However, isolation and structural characterization of tertiary cell wall RG-I have been performed only for three plant species—flax [12,19], hemp [8], and aspen [20]. In addition to the general similarities of polymers from all three species, i.e., RG-I with long side chains from β-(1,4)-galactans, some structural peculiarities and differences in the pattern of immunolabeling were noted [8]. The variations in fiber-specific RG-I structure and/or in the parameters of the enzymes involved in its modification may be coupled to the reported differences in stiffness and apparent elastic moduli of tertiary cell walls in fibers from different plant species [2].

To confirm the importance of RG-I for tertiary cell wall function and to understand both the general and species-specific parameters of these polymers, it is necessary to widen the list of deeply characterized RG-I from the fibers of various plants. It is known that species of the Fabaceae are characterized by the formation of gelatinous fibers in various organs [17,21,22]. Fibers with a gelatinous cell wall have been described in Fabaceae plants with root contraction [23,24], stem undulation and contraction [25], and inclined seedlings [26]. However, the presence of RG-I has not been checked in the fibers of Fabaceae plants. For in-depth biochemical characterization, we chose the phloem fibers of the *Phaseolus vulgaris* stem, since these fibers are gathered in bundles and can be purified from other stem tissues. Together with that, we describe tertiary cell wall immunolabeling by RG-I-related antibodies in fibers from several species of Malphigiales, Fabales, and Rosales. The obtained results confirm RG-I as an ever-present polymer of tertiary cell walls and suggest that the differences in its structure in various plants may be taxonomically related.

## 2. Results

### 2.1. Microscopy and Immunohistochemistry of the Phloem Fiber Cell Wall in Phaseolus vulgaris Stem

**Figure 1:**
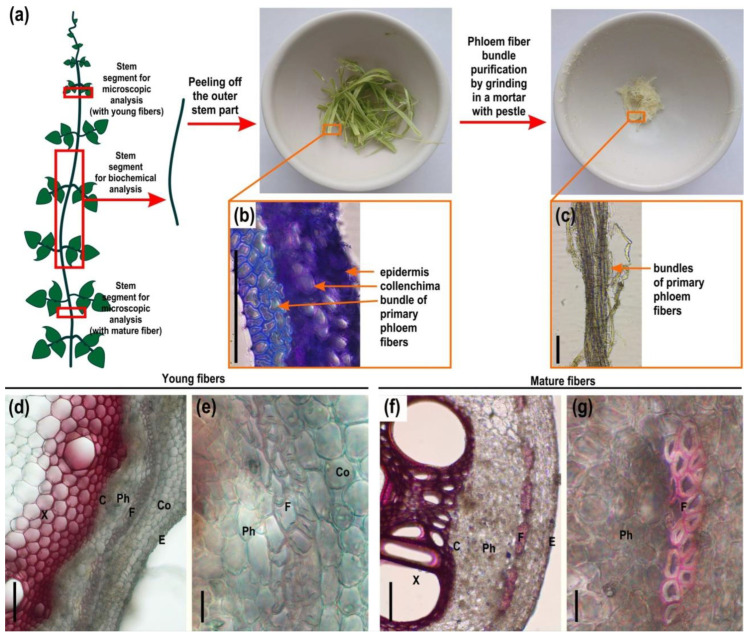
(**a**) Scheme of sample collection from common bean (*Phaseolus vulgaris*) stem for microscopy and biochemical analysis (red boxes); (**b**) cross-section of outer stem part, stained by toluidine blue; and (**c**) isolated phloem fiber bundles. Cross-sections of (**d**,**e**) young (with young phloem fibers) and (**f**,**g**) mature (with mature phloem fibers) part of the stem of a common bean stained by phloroglucinol-HCl. In young fibers the cell wall is not lignified. In mature fibers, only the outer layers of the cell wall undergo lignification, while the tertiary cell wall remains unlignified. C—cambium, Co—collenchyma, E—epidermis, F—fibers, Ph—phloem, X—xylem. Scale bar (**b**–**d**,**f**) 100 µm; (**e**,**g**) 20 µm.

**Figure 2:**
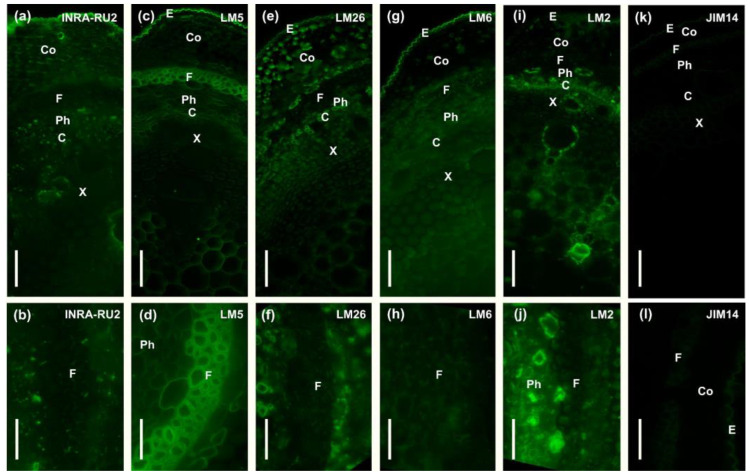
Immunolabeling of common bean stem cross-sections with monoclonal antibodies (**a**,**b**) INRA-RU2, specific for rhamnogalacturonan I backbone; (**c**,**d**) LM5, specific for linear β-(1,4)-d-galactan; (**e**,**f**) LM 26 specific for branched β-(1,4)-d-galactan; (**g**,**h**) LM6, specific for linear α-(1,5)-l-arabinan; (**i**,**j**) LM2; and (**k**,**l**) JIM14, specific for arabinogalactan protein. C—cambium, Co—collenchyma, E—epidermis, F—fibers, Ph—phloem, X—xylem. Scale bar (**a**,**c**,**e**,**g**,**i,k**) 100 µm; (**b**,**d**,**f**,**h**,**j**,**l**) 50 µm.

**Figure 3:**
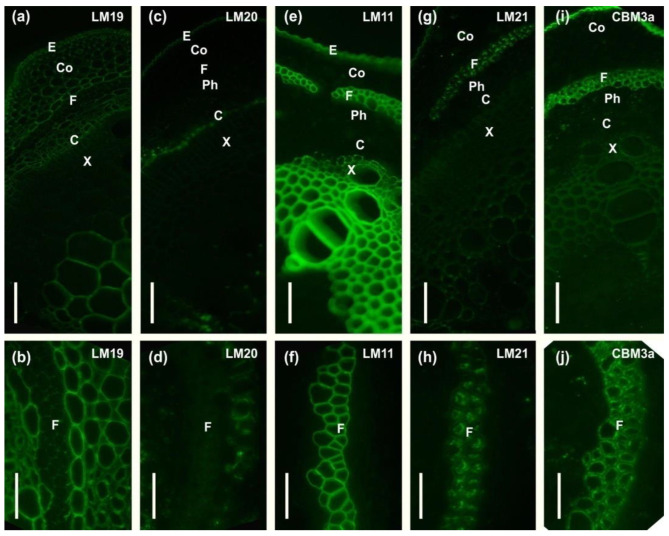
Immunolabeling of common bean stem cross-sections with monoclonal antibodies (**a**,**b**) LM19 and (**c**,**d**) LM20, specific for homogalacturonan with low and high levels of esterification. (**e**,**f**) LM11, specific to β-(1,4)-d-xylan; (**g**,**h**) LM21, specific for (galacto)(gluco)mannan; and (**i**,**j**) carbohydrate-binding module CBM3a that specifically recognizes the planar surface of crystalline cellulose. C—cambium, Co—collenchyma, E—epidermis, F—fibers, Ph—phloem, X—xylem. Scale bar (**a**,**c**,**e**,**g**,**i**) 100 µm; (**b**,**d**,**f**,**h**,**j**) 50 µm.

The upper and lower parts of the common bean (*Phaseolus vulgaris* L.) stem were analyzed by light microscopy, and the middle part of the stem was analyzed through immunohistochemical methods (Figure 1, Figure 2 and Figure 3). In the common bean stem, the primary phloem fibers were grouped in bundles, surrounded by a layer of parenchyma cells and located in the cortex between several layers of collenchyma cells and sieve tube elements of the phloem (Figure 1d,f). Although the fiber bundles in the common bean stem were formed by only 2–3 layers of cells, they looked dense and formed a strand along the entire length of the stem, permitting their isolation (Figure 1c). Phloroglucinol-HCl staining of stem sections and comparison with highly lignified xylem tissues indicated that the cell walls of phloem fibers contained some phenolic components only in the outer layers of the cell wall and only at a mature stage of fiber development (Figure 1f,g). The inner layer of the phloem fiber cell walls was not lignified in young and mature stems (Figure 1e,g).

The cell wall composition of phloem fibers of common bean stems was characterized using antibodies specific for the rhamnogalacturonan I backbone (INRA-RU2), and its side chains from linear and branched β-(1,4)-d-galactan (LM5 and LM26) or α-(1,5)-l-arabinan (LM6), arabinogalactan protein (LM2, JIM14), low- and high-esterified homogalacturonan (LM19, LM20), β-(1,4)-d-xylan (LM11), heteromannan (LM21), and carbohydrate binding module (CBM3a) that specifically recognizes the planar surface of crystalline cellulose (Table 1, Figure 2 and Figure 3).

In the cell wall of common bean fibers, epitopes to the antibody specific for β-(1,4)-d-galactan (LM5) were distributed throughout the thickness of the inner layer (tertiary cell wall), whereas they were absent in the outer layers, the middle lamella, and the primary and secondary cell walls (Figure 2c,d). Epitopes of the backbone of RG-I itself (INRA-RU2) and other possible constituents of its side chains, like branched galactan (LM26) or arabinan (LM6), were not detected in the cell walls of *Phaseolus vulgaris* fibers (Figure 2a,b,e–h).

Epitopes for antibodies specific for arabinogalactan protein (LM2, JIM14) and homogalacturonan (LM19, LM20) were not found in the cell walls of common bean fibers (Figure 2i–l and Figure 3a–d). In phloem fibers, epitopes for the xylan-specific antibody (LM11) were detected only in the thin outer layer of the cell wall (Figure 3e,f). Xylem tissues, as expected, were heavily labeled by LM11 (Figure 3e). Epitopes for antibodies specific for mannans (LM21) were present in the inner layers of the fiber cell wall (Figure 3g,h).

The most active, as compared with other tissues, fluorescence signal from bound CBM3a was observed in phloem fibers, indicating their enrichment in crystalline cellulose (Figure 3i,j). Some labeling by CBM3a was also observed in other tissues, including the xylem and epidermis.

### 2.2. Cell Wall Composition of Phaseolus vulgaris Fibers

**Figure 4:**
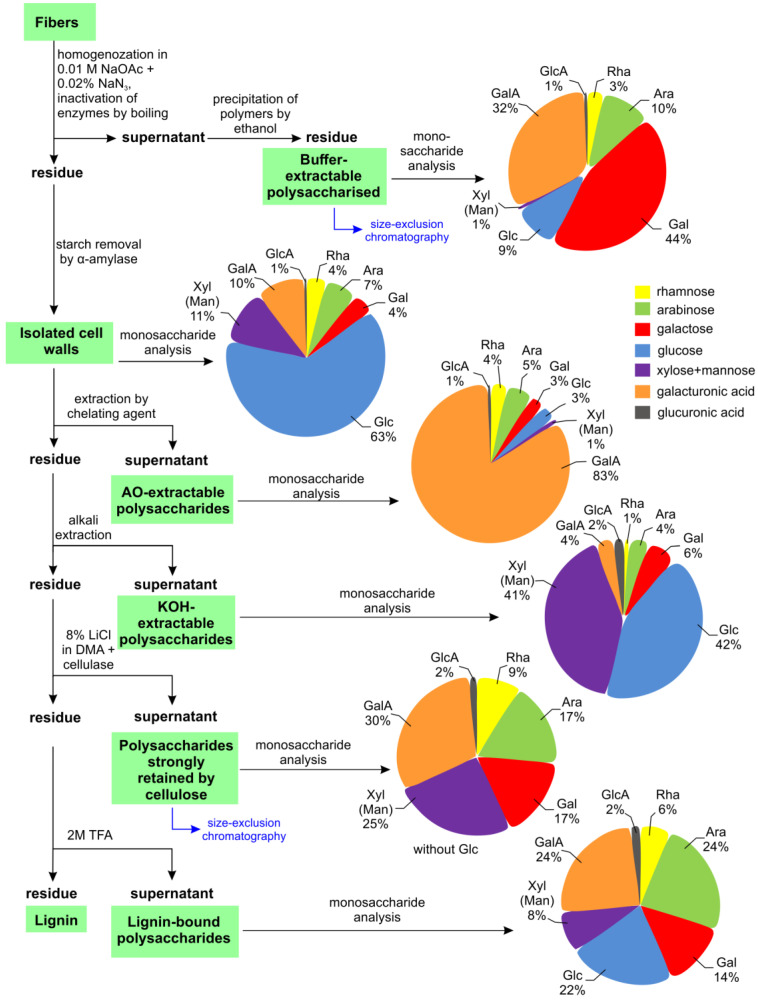
Scheme of extraction of the cell wall fractions of common bean fibers with their monosaccharide proportion (mol%).

For the analysis of polysaccharides of different cell wall fractions of the primary phloem fibers of common bean stem, we used fibers washed from adjacent tissues by grinding in a mortar with a pestle in 80% ethanol (Figure 1a–c). To characterize the cell wall polymers more specifically, the fiber cell wall was fractionated into buffer-extractable polysaccharides, ammonium oxalate AO-extractable polysaccharides, 4 M KOH-extractable polysaccharides, polysaccharides strongly retained by cellulose, lignin-bound polysaccharides, and lignin via the sequential extraction procedure shown in Figure 4.

The predominant cell wall component of common bean fibers was cellulose, which accounted for 74.7% of the dry weight of the cell wall (Table 2). Glucose, part of which belongs to amorphous cellulose, xylose, and galacturonic acid, predominated in the composition of TFA-hydrolysable polysaccharides. Arabinose, rhamnose, and galactose also comprised significant proportions.

Non-cellulosic polymers accounted for approximately 20% of the cell wall dry weight, as determined by the total yields of AO- and KOH-extractable fractions and polysaccharides strongly retained by cellulose (excluding glucose) and lignin (Table 2). Xylose (mannose) and glucose-containing polymers predominated in the KOH-extractable fraction; some of these polymers were also released by AO, but the major components of the latter were pectin polysaccharides, consisting of galacturonic acid, rhamnose, arabinose, and galactose (Figure 4).

### 2.3. Polymers Extracted with Buffer and Obtained after Complete Cellulose Destruction from the Cell Wall of Phaseolus vulgaris Fibers

The buffer-extracted fraction contained polysaccharides weakly bound to the cell wall and polysaccharides in the process of biosynthesis and incorporation into the cell wall. Complete destruction of cellulose by dissolving the alkali-unextractable pellets in DMA with LiCl, followed by cellulase treatment, resulted in the release of polysaccharides strongly retained by cellulose.

**Figure 5:**
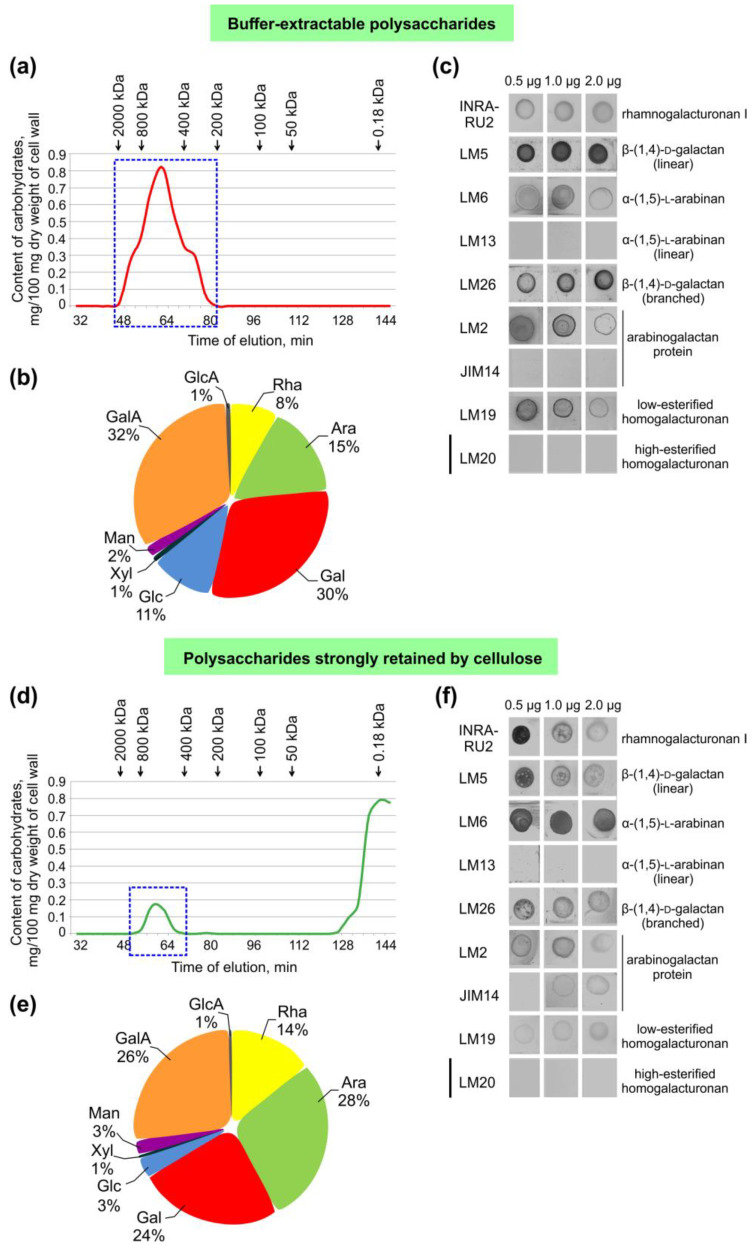
Analysis of high-molecular-weight subfractions of (**a**–**c**) buffer-extractable and (**d**–**f**) strongly retained by cellulose polysaccharides, isolated from the cell wall of common bean fibers. Elution profile of (**a**) buffer-extractable and (**d**) strongly retained by cellulose polysaccharides obtained after size-exclusion chromatography on a Sepharose CL-4B with indication of the high-molecular-weight subfraction (marked with a blue dotted line); (**b**,**e**) proportions of monosaccharides obtained after TFA hydrolysis; and (**c**,**f**) immunodot analysis of high-molecular-weight subfractions. Scale bar (**c**,**f**) 0.5 mm.

Galactose, galacturonic acid, arabinose, and rhamnose were the major monosaccharides present in the composition of buffer-extractable fractions of common bean fibers (Figure 4). The polymers of this fraction, purified by precipitation in ethanol, were eluted in the range of 200–2000 kD (Figure 5a). GalA and Gal were the major monomers of the polymer fraction; among the other major monomers, Ara, Glc, and Rha were also expressed. A GalA/Rha ratio greater than one indicates the presence of polygalacturonic acid in the pectin components of the fraction. The proportion of polygalacturonic part f in the backbone of the pectic polymer constituted 60% of all pectic backbones (Figure 5b). The presence of rhamnose and monomers specific for galactans, arabinans, and arabinogalactans indicated the presence of RG-I in this fraction (Figure 5b).

Immunolabeling of the buffer-extractable fraction with the INRA-RU2 antibody suggested the presence of RG-I (Figure 5c). Intensive binding of LM5 revealed the presence of linear β-(1,4)-galactan. Labeling by LM26 indicated the presence of branched β-(1,4)-galactan in addition to the linear one. Application of LM6 indicated the presence of α-(1,5)-arabinan, but there was no labeling by LM13, another antibody specific for linear α-(1,5)-arabinan. The binding of the LM2 antibody indicated the presence of arabinogalactan proteins. Immunolabeling with LM19 suggested the presence of low-esterified homogalacturonan in the buffer-soluble fraction of the common bean fiber cell wall. However, there was no immunolabeling with LM20, an antibody specific for high-esterified homogalacturonan.

Separation of the polymers isolated from the alkali-unextractable pellets by dissolution with LiCl in DMA followed by cellulase treatment by gel filtration also revealed high-molecular-weight polysaccharides eluted in the range 400–2000 kD; other components of this fraction eluted in the range less than 30 kD (Vi) (Figure 5d). In the high-molecular-weight subfraction, Ara, Gal, GalA, and Rha predominated, suggesting the presence of RG-I (Figure 5e). The GalA/Rha ratio in this fraction was two-times lower than in the composition of the high-molecular-weight subfraction of buffer-extractable polysaccharides, but galacturonic acid also predominated over rhamnose; the proportion of homogalacturonan accounted for 32% of the total pectic backbones (Figure 5b,e).

The main features of the polysaccharides released after cellulose degradation were more intense binding of INRA-RU2 and LM6 antibodies specific for RG-I backbone and α-(1,5)-arabinan, respectively, and less intense immunolabeling by LM5 and LM26 specific for linear and branched β-(1,4)-galactan (Figure 5c,f). As in the buffer-extractable fraction, there was no immunolabeling with the LM13, specific for linear α-(1,5)-arabinan. There was also no immunolabeling with the LM20 antibody, specific for high-esterified homogalacturonan. Immunolabeling with antibodies specific for low-esterified homogalacturonan (LM19) was of extremely low intensity. A weak and extremely weak intensity of immunolabeling was observed with antibodies specific for arabinogalactan (LM2, JIM14).

**Figure 6:**
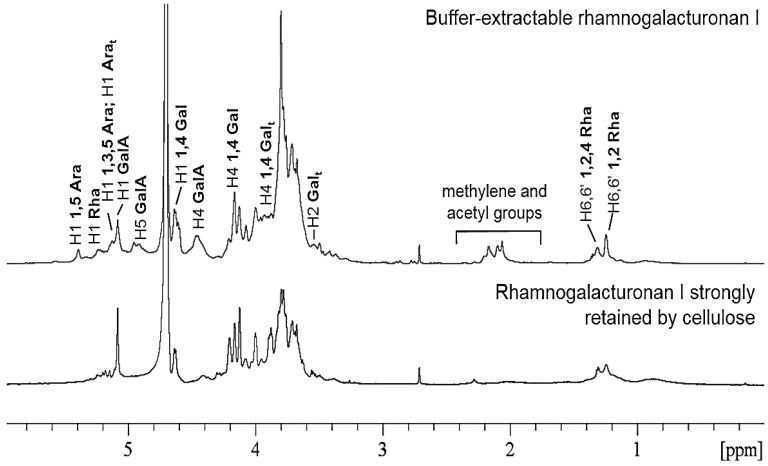
^1^H NMR spectra of rhamnogalacturonans I extracted by buffer and obtained after total destruction of the cellulose of the fiber cell wall from common bean.

The results of the immunodot analysis were confirmed by NMR data. Analysis of the buffer extracted and obtained after complete destruction of the cellulose polysaccharides by ^1^H NMR spectroscopy revealed the signals of non-equivalent protons of methyl groups in the high-field and anomeric protons in the weak field related to rhamnosyl residues, unsubstituted (1.24 and 5.23 ppm) and substituted by side chains (1.31 and 5.23 ppm), as well as galacturonic acid (H1 5.03, 5.05, 5.09 ppm, and H4 4.43 ppm) related to both RG-I and homogalacturonan (Figure 6). This confirms the presence of RG-I in these fractions. The ratio of the integral intensities of the signals of the methyl groups at 1.24 and 1.31 ppm demonstrates that about 46% of the rhamnosyl residues of both the buffer-extractable and the cell wall RG-I were substituted by side chains. This index is lower than that of flax fiber RG-I, in which about 70% of the backbone rhamnose is substituted by side chains [12] but is very close to the degree of substitution of the backbone of hemp fiber RG-I, in which 48% of the rhamnose is substituted by side chains [8]. Signals in the range of 4.60–4.64 ppm and 4.16 ppm indicate the presence of β-(1,4)-galactans in the sample [12,41,42]. This type of side chain has been previously observed in rhamnogalacturonans I of the tertiary cell walls of other plants [18,20,43]. The signals at 5.39, 5.14, and 5.10 ppm suggest the presence in the structure of polysaccharides from common bean fibers, together with β-(1,4)-galactans, α-(1,5)-, and α-(1,3,5)-arabinans (more pronounced for the fraction of buffer-extractable polymers, Figure 6), which we have also previously observed for RG-I isolated from hemp fibers [8]. In the case of a polysaccharide strongly retained by cellulose in the cell wall of common bean fibers, the major part of the arabinose-containing chains was represented mainly by terminal residues (5.14 ppm) and 1,3,5-substituted chains (5.10 ppm) [44,45].

**Figure 7:**
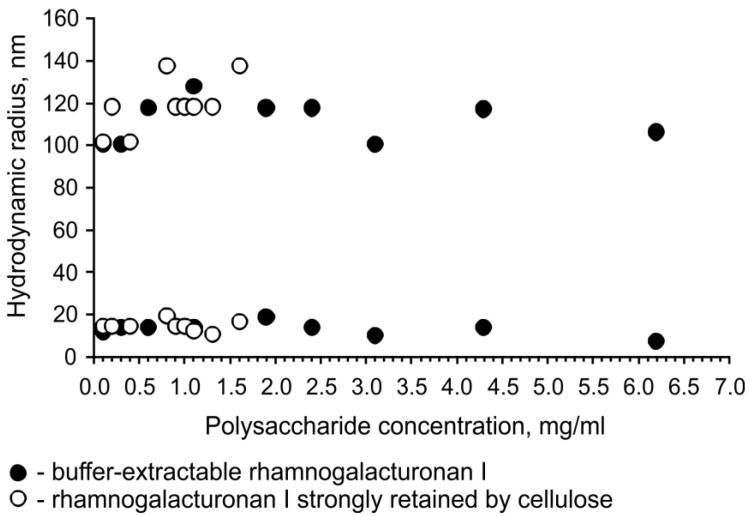
Concentration dependence of the hydrodynamic radii of small and large particles of rhamnogalacturonans I extracted by buffer (black circles) and obtained after total destruction of cellulose (white circles) from the cell wall of common bean fibers.

Analysis of the hydrodynamic behavior of rhamnogalacturonans I from common bean fibers using dynamic light scattering revealed that, for both types of polysaccharide, there were two types of particles in solution at all concentrations analyzed. The hydrodynamic radius of the small particles varied in the range of 10 to 20 nm; the radius of the large particles, possibly represented by aggregates of molecules, was about 100–130 nm (Figure 7). All particles in the samples demonstrated a similar distribution of relaxation times, irrespective of the concentration of polysaccharides in the solution. Dilution of the polysaccharide to 0.1 mg/mL did not change the diffusion rate of large particles, which may indicate either the presence of two types of polysaccharides with different chain lengths and/or conformations or the stability of molecular aggregates in water at dilution. The latter seems more likely, as large particles of flax fiber RG-I with long side chains also demonstrate the stability of aggregates upon dilution and analysis in water. These aggregates were formed by galactan chains, and the subsequent transition from large to small particles was only detected after shortening the galactan chains and at low concentrations of the polysaccharide in solution [19].

### 2.4. Immunohistochemistry of Fiber Cell Walls in Other Species of Fabacea and in Species of Some Other Families

**Figure 8:**
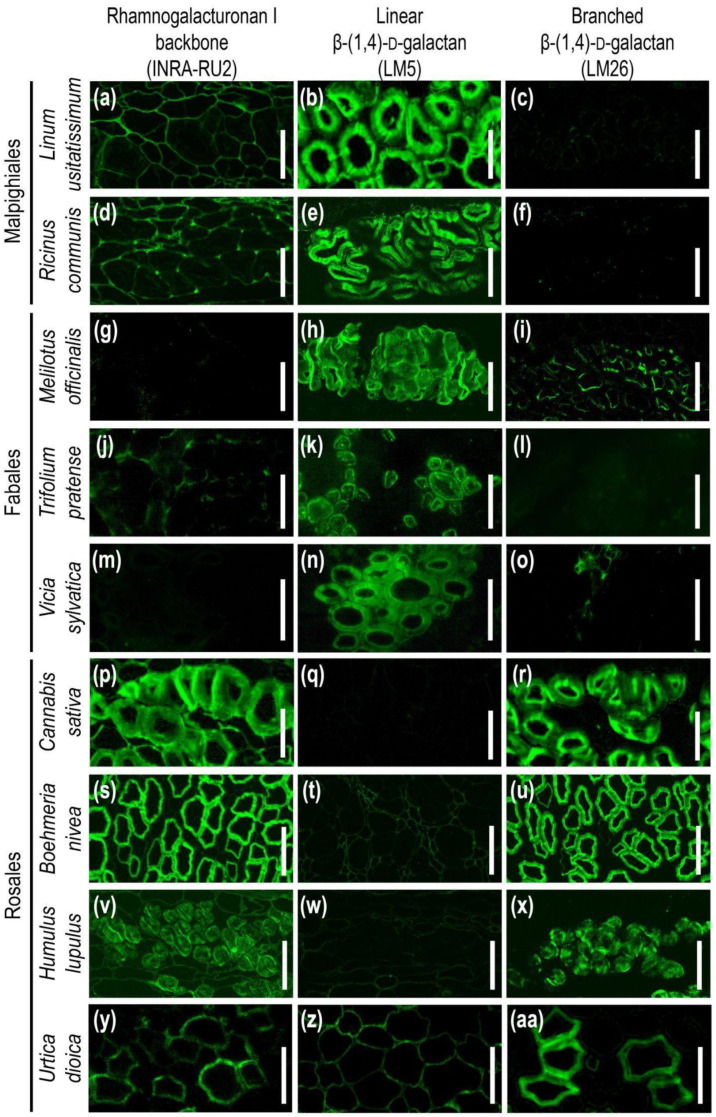
Immunolabeling of developing fibers with tertiary cell walls on stem cross-sections of different plant species belonging to Malpighiales, Fabales, and Rosales orders with monoclonal antibodies (**a**,**d**,**g**,**j**,**m**,**p**,**s**,**v**,**y**) INRA-RU2; (**b**,**e**,**h**,**k**,**n**,**q**,**t**,**w**,**z**) LM5; and (**c**,**f**,**i**,**l**,**o**,**r**,**u**,**x**,**aa**) LM26. Scale bar (**a**–**x**) 50 µm; (**y**–**aa**) 20 µm.

Immunohistochemical analysis of fiber cell walls in other species was carried out on cross-sections of the middle parts of the growing stems of several members of the orders Malpighiales, Fabales, and Rosales (Figure 8). Thin sections of dehydrated and resin-embedded stem segments were immunolabeled with antibodies specific for the rhamnogalacturonan I backbone (INRA-RU2) and its linear (LM5) and branched (LM26) β-(1,4)-d-galactan side chains and then observed by confocal laser scanning or epifluorescence microscopy. Flax (*Linum usitatissimum*) and castor bean (*Ricinus communis*) were analyzed as Malpighiales species. In addition to the common bean, other Fabales species were also studied—sweet clover (*Melilotus officinalis*), clover (*Trifolium pratense*), and wood vetch (*Vicia sylvatica*). Hemp (*Cannabis sativa*), ramie (*Boehmeria nivea*), hops (*Humulus lupulus*), and nettle (*Urtica dioica*) were analyzed as representatives of the Rosales.

According to the immunohistochemical results, the tertiary cell walls of fibers from Malpighiales species, flax and castor bean, did not expose epitopes for the INRA-RU2 and LM26 antibodies, which are specific for the backbone of rhamnogalacturonan I and branched β-(1,4)-d-galactan as their side chains, respectively (Figure 8a,c,d,f). At the same time, epitopes to the LM5 antibody specific for linear β-(1,4)-d-galactan were heavily abundant in the developing tertiary cell wall of fibers from Malpighiales species (Figure 8b,e). The same different presentation of LM5 and INRA-RU1 epitopes has been reported in developing G-layers in tension wood of poplar, another representative of Malphigiales [7].

A similar picture was observed for the distribution of these antibodies in the fiber cell walls of Fabales species. Epitopes to the antibody specific for rhamnogalacturonan I backbone (INRA-RU2) were absent in the developing tertiary cell wall of common bean, sweet clover, clover, and wood vetch fibers (Figure 2a,b and Figure 8g,j,m). Epitopes to the antibody for branched galactan (LM26) were also absent in the fiber cell wall of common bean, clover, and wood vetch (Figure 2e,f and Figure 8l,o). The exception was sweet clover, where the presence of epitopes for the LM26 antibody was observed in the thin innermost layer of the tertiary cell wall (Figure 8i). Epitopes to the LM5 antibody, specific for linear β-(1,4)-d-galactan, were present in the fiber cell walls of all analyzed Fabales species (Figure 2c,d and Figure 8h,k,n).

The species of the Rosales formed a somewhat isolated group (Figure 8p–aa). The developing tertiary cell wall of all four representatives of this taxon had epitopes for the INRA-RU2 antibody, specific for the rhamnogalacturonan I backbone (Figure 8p,s,v,y). In addition, the tertiary cell wall of these species was also actively immunolabeled with an LM26 antibody specific for branched β-(1,4)-d-galactan (Figure 8r,u,x,aa). At the same time, epitopes to LM5, specific for linear β-(1,4)-d-galactan, were absent in the developing tertiary cell walls of hemp, ramie, hops, and nettle fibers (Figure 8q,t,w,z).

### 2.5. β-Galactosidase Activity in Tertiary Cell Walls of Common Bean and Some Other Plant Species

**Figure 9:**
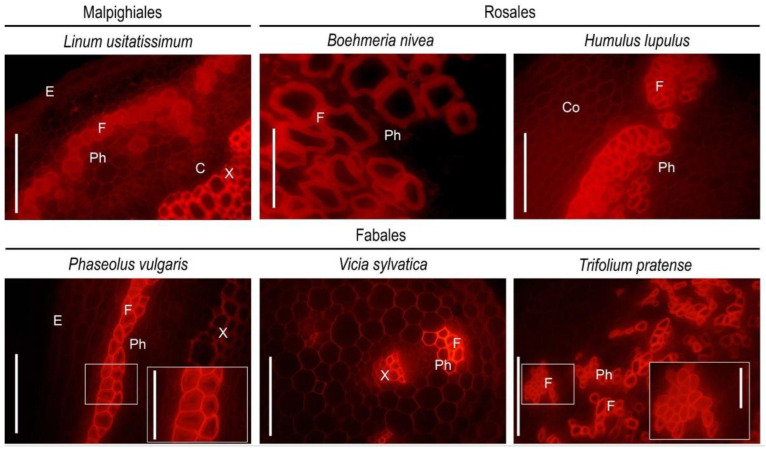
β-d-galactosidase activity in the stems of flax (*Linum usitatissimum*), ramie (*Boehmeria nivea*), hops (*Humulus lupulus*), and common bean (*Phaseolus vulgaris*); tendrils of wood vetch (*Vicia sylvatica*); and storage root of clover (*Trifolium pratense*). In inserts, a larger image of the fibers are given. C—cambium, Co—collenchyma, E—epidermis, F—fibers, Ph—phloem, X—xylem. Bars are 100 µm for main figures and 50 µm for insets.

To assess the presence of galactosidase activity in the tertiary cell wall of fibers, transverse sections of flax (*Linum usitatissimum*), hops (*Humulus lupulus*), and common bean (*Phaseolus vulgaris*) stems, as well as wood vetch (*Vicia sylvatica*) tendrils and clover (*Trifolium pratense*) storage root, were stained with the fluorogenic substrate resorufin-d-galactopyranoside (Figure 9). No staining was observed in the control sections, which were heated to 90 °C for 5 min in a water drop before substrate addition. The highest activity in all samples was detected in the secondary cell walls of both phloem and xylem fibers. However, the tertiary cell walls of all samples also had galactosidase activity throughout the whole thickness.

## 3. Discussion

### 3.1. Phloem Fibers of Phaseolus vulgaris Stem Deposit Tertiary Cell Wall

The stem of *Phaseolus vulgaris* contains bundles of phloem fibers with thickened cell walls, well resolved by light microscopy (Figure 1d–g). As distinct from the walls of xylem cells that are intensively stained by phloroglucinol-HCl used to reveal lignin, the cell walls of phloem fibers are barely lignified (Figure 1d–g). Even at an advanced stage of development, only the outer layer of the fiber cell wall was stained, rather slightly in comparison to xylem, while the inner layer remained clear. The stained layer corresponds to the one labeled by the anti-xylan antibody LM11 (Figure 3e,f). Thus, part of the phloem fiber cell wall is represented by the secondary cell wall, whose distinctive feature in angiosperms is the presence of xylan and lignin. However, the inner part of the thickened cell wall in phloem fibers has a different composition. In addition to the absence of lignin and xylan, it is characterized by the labeling of this layer by an LM5 antibody specific for β-(1,4)-galactans, the side chains of RG-I (Figure 2c,d). The inner layer meets the criteria suggested by Gorshkova et al. [1] and Chery et al. [3] to detect the tertiary cell wall, also often designated as the G-layer of the secondary cell wall. Thus, the cell walls of phloem fibers in *Phaseolus vulgaris* have, in addition to the thin middle lamella and primary cell wall, two major layers: one closer to the periphery secondary cell wall and the inner thickest layer, the tertiary cell wall. The proportions of the secondary and tertiary cell walls in common bean differ from the classical examples, like primary phloem fibers of flax or hemp, where the tertiary cell wall highly predominates [8], but the major characteristics are the same—absence of xylan and lignin, presence of RG-I in the tertiary cell wall, and the opposite situation in the secondary cell wall.

The possibility of isolating phloem fibers from the developing stem of the common bean permitted biochemical characterization of the walls of the particular cell type and their major constituents. The walls of phloem fibers contain some lignin and xylan (Figure 4, Table 2) coming from the secondary cell wall layer, which, in angiosperms, mainly consists of xylan, lignin, and cellulose present in roughly equal proportions [5]. However, isolated phloem fibers of common bean have a much higher proportion of cellulose, which is due to the highly cellulosic tertiary cell wall layer, and contain significant amounts of RG-I (Figure 4, Table 2). Moreover, RG-I with side chains made of β-(1,4)-galactans is present in the buffer-extractable fraction and constitutes the high-molecular-mass peak, as confirmed by dot-blot analysis (Figure 5a–c) and NMR (Figure 6). Such polymers are specific for samples containing fibers depositing tertiary cell walls in flax [10] and are also observed in hemp bast fibers [8], aspen tension wood [20], and isolated G-layers of poplar tension wood [7]. Together with that, the phloem fibers of common bean have pronounced β-galactosidase activity (Figure 9), which has been proven to be important for tertiary cell wall deposition in flax [13] and is also abundant in the fibers of aspen tension wood [20]. The obtained results allow us to classify the major part of the fiber cell wall of *Phaseolus vulgaris* as the tertiary cell wall and describe its similarities and differences with other plant species.

### 3.2. Structural Details of Rhamnogalacturonan I in Gelatinous Fibers of Various Species May Differ

RG-I was detected in fibers at the stage of tertiary cell wall formation in all biochemically analyzed plant species. A key feature of this polysaccharide from the tertiary cell wall is the presence of galactan side chains. However, both the structural features of these chains and other structural parameters of polymer may vary to some extent depending on the source of the fibers.

Flax fiber RG-I is the most studied variant of the tertiary cell wall RG-I. It is significantly distinguished from most other fiber rhamnogalacturonans I by the absence of homogalacturonan (HG) in the backbone [19], the highest degree of substitution of rhamnosyl residues with galactan side chains (more than 70% of all rhamnose), and an extremely low proportion of non-galactosyl residues in the side-chain structure [12].

Rhamnogalacturonans I, strongly retained by cellulose in the tertiary cell walls of other fiber sources, are more similar to each other, and the common bean RG-I structure is very similar to them. These polysaccharides contain HG fragments, the proportion of which can vary and reach 20% from the pectic backbone in aspen tension wood [20], 32% in common bean fibers, and 33 and 41% in primary and secondary hemp fibers, respectively [8]. Before incorporation into the cell wall, the proportion of HG in all variants of RG-containing fractions is always higher than after entering the fraction of polymers strongly retained by cellulose [8,11,20]. Along with galactans, common bean fiber RG-I, as well as polysaccharides from hemp and aspen tension wood fibers, include arabinose-containing components (arabinogalactans and/or arabinans), which also distinguish them from flax fiber RG-I.

According to the NMR data, the degree of substitution of the rhamnosyl residues of the backbone in common bean RG-I is about 46% of all rhamnosyl residues; that is comparable to the RG-I from aspen tension wood (47%) [20], slightly lower than in the RG-I variants from hemp (52%) [8] and significantly lower than in the RG-I from flax fibers (72%) [12]. In most objects, this value for RG-I does not change after incorporation into the cell wall. This suggests that the total removal of side chains does not occur in muro. At the same time, shortening of the galactan chains into the cell wall is characteristic of all types of analyzed RGs-I fiber. Judging by the Gal/Rha ratio, which characterizes the average length of the galactan side chains, they remain the shortest in the case of RGs-I of hemp [8] and common bean fibers (Gal/Rha less than 2). In the RG-I of flax fibers and aspen tension wood, the average length of the chains is almost twice as large (Gal/Rha 3.8 and 3.6) [12,20].

Despite some structural differences (HG in the backbone and the presence of arabinose-containing side chains), the RG-I of common bean fibers has similar hydrodynamic behavior to the RG-I of flax fibers and, like this polysaccharide, is capable of forming strong molecular aggregates in water that are not destroyed when the polysaccharide concentration decreases. The hydrodynamic radius of the aggregates formed by these polysaccharides is close and varies for common bean RG-I from 100 to 130 nm and for flax RG-I from 140 to 190 nm [19]. This is more than three-times smaller than the size of the aggregates formed in water by rhamnogalacturonans from flaxseed mucilage, characterized by a different position of rhamnose substitution (at *O*-3 instead of *O*-4), extremely short side chains, and the presence of both HG fragments and homorhamnan in the backbone [46]. But this size is, for example, almost twice as large as the size of aggregates formed in water by mixed-linkage glucan with flexible kinks in the structure of the chain [47]. The decrease in the length of the galactan side chains of common bean fiber RG-I after incorporation into the cell wall, as in the case of flax RG-I [19], is not accompanied by a decrease in the hydrodynamic radii of aggregates of these polysaccharides (Figure 7).

RG-I-rich pectins have a rather flexible geometry, with a persistence length much inferior to that of the rigid rod-like HG [48,49,50] and close to that of amylopectin, one of the most flexible polysaccharides. The removal of neutral side chains and reduction in branching do not significantly affect the average persistence length and flexibility of the remaining backbone. This was demonstrated with isolated sugar beet RG-I domains [49], suggesting that the presence of the RG-I itself already contributes to increased flexibility of the pectin. Moreover, the presence of side chains of arabinan, galactan, and/or arabinogalactan in the structure of pectin, which, along with RG-I, also contains significant inclusions of HG domains, contributes to additional coil-up of the molecule. Combination of such structural domains leads to the formation of very compact or spherical structures with a lower intrinsic viscosity compared to elongated rigid rod-shape pure HG and pectins with a high HG content and similar molecular weights [51,52].

Thus, when choosing a compact polysaccharide suitable for the realization of the mechanism of tension creation in the tertiary cell wall, which is based on the capture of RG-I by laterally interacting cellulose microfibrils [1], two strategies can be used: (1) selection of pure flexible RG-I with one type of side chain—galactans, and the possibility of regulating their length during cell wall maturation using tissue-specific β-galactosidase (as is implemented in flax fibers). The presence of 1,4,6-Gal in the side-chain structure in this case can act as an additional regulator that limits the action of the enzyme and allows part of the long chains to be left untruncated; (2) the choice of pectin polysaccharide based on RG-I with significant inclusions in the structure of the HG backbone, and at the same time with the additional arabinose-containing elements in the side chains. The presence of HG blocks largely reduces the affinity for cellulose. Arabinose-containing side chains increase the flexibility of the molecules, apparently contributing not only to their folding but also to their self-aggregation.

### 3.3. Rhamnogalacturonan I with Side Chains of β-(1,4)-Galactan as an Ubiquitous Polymer of Tertiary Cell Wall in Various Species

Analysis of the immunolabeling with RG-I-related antibodies of tertiary cell walls of developing fibers in several species from three different orders (Malphigiales, Fabales, and Rosales) of dicotyledonous plants revealed the presence of the RG-I in all of them (Figure 8). RG-I backbone and/or β-(1,4)- and β-(1,4,6)-galactan were detected by corresponding antibodies in the G-layers of fibers in several other species [7,15,16,17,18,53]. Various biochemical techniques, like NMR spectroscopy, mass spectrometry, immunodot analysis, and polysaccharide analysis using carbohydrate gel electrophoresis (PACE), applied to polysaccharides extracted from fibers depositing tertiary cell walls always confirm the presence of the RG-I backbone and side chains made of β-(1,4)-galactans [7,8,12,19,20] in the current paper. Thus, RG-I can be considered the ever-present polymer of the tertiary cell wall and used as a marker to distinguish the tertiary cell wall from the preceding secondary cell wall.

**Figure 10:**
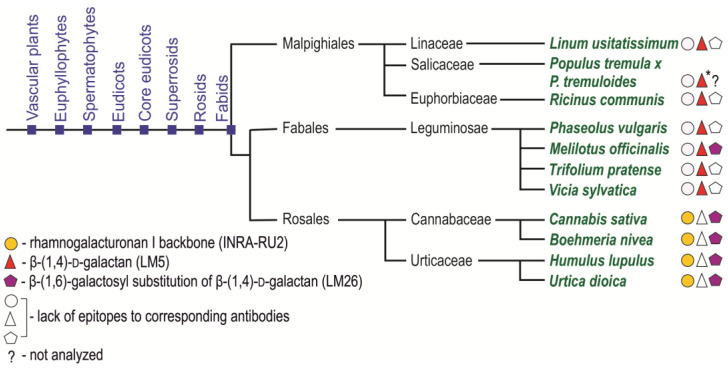
Analyzed species of Malpighiales, Fabales and Rosales, their phylogeny (following [57]) and summary of the distribution of epitopes to antibodies specific to the rhamnogalacturonan I backbone (INRA-RU2), β-(1,4)-d-galactan (LM5), and β-(1,6)-galactosyl substitution of β-(1,4)-d-galactan (LM26) in tertiary cell walls of their developing fibers. * Data for *Populus* spp. are given according to [7].

However, immunolabeling in situ, using the same antibodies as in immunodot analysis of isolated polymers, often reveals either the backbone or the side chains only, at least in developing fibers at the stage of active tertiary cell wall deposition. For example, flax fibers are heavily labeled by the LM5 antibody but do not bind the INRA-RU2 antibody (Figure 9). As opposed to that, hemp fibers are intensively labeled by INRA-RU2 but do not bind the LM5 antibody [8] (Figure 8). The absence of LM5 binding to the phloem fiber cell wall in hemp has also been described [54,55]. The pattern observed in hemp can be explained by the high branching degree of β-(1,4)-galactan chains that is revealed by NMR analysis [8] and restricts binding of the LM5 antibody that has been raised against the linear pentamer [28]. Indeed, LM26 antibodies specific for branched β-(1,4,6)-galactan [29] bind the tertiary cell walls of hemp fibers quite well (Figure 8). However, the situation is more complicated since the structure of RG-I in hemp resembles that in common bean, but the profile of tertiary cell wall immunolabeling in common bean is closer to that in flax (Figure 8). A similar pattern with the presence of intensive immunolabeling by INRA-RU1 and INRA-RU2 antibodies, both of which recognize the RG-I backbone [27], and the absence of signal from LM5 throughout the G-layer was observed by confocal and transmission electron microscopy in the stinging nettle, which belongs to Rosales [56] (Figure 8). Opposite to that, developing G-layers in tension wood of poplar (Malphigiales) bind the LM5 but not the INRA-RU1 antibody [7]. This means that the differences in in situ immunolabeling of tertiary cell walls by RG-I-related antibodies can be taxonomically related since the species from the same order have similar patterns (Figure 10). It should be mentioned that in the course of fiber cell wall maturation, the described pattern can become less sharp, as can be seen in [7], and this can be related to the RG-I processing by cell-wall-located enzymes [1].

Altogether, the RG-I in muro metabolism is in the center of tertiary cell wall development and maturation. The peculiarities of the presentation of various RG-I epitopes at the tissue cross-sections may reflect, together with the specific details of the polymer structure, the peculiarities of the supramolecular organization of the polymer, the same as the differences in interactions of the RG-I backbone and side chains with other cell wall polymers. Further studies should be aimed to analyze the consequences of the differences in RG-I for the function of tertiary cell wall in planta, like the force generation in coiling tendrils [4,58] and for the properties of technical fibers obtained from fiber crops [18]. The structure and characteristics of tertiary cell wall constituents should be considered while discussing the prospects of artificial actuation systems inspired by the principles of plant fiber construction; such systems are becoming quite popular in robotic devices [2,59].

## 4. Materials and Methods

### 4.1. Plant Material

Plants of common bean (*Phaseolus vulgaris* L., cultivar Fantasia), flax (*Linum usitatissimum* L.), castor bean (*Ricinus communis* L.), ramie (*Boehmeria nivea* (L.) Gaudich.), and clover (*Trifolium pratense* L.) were grown outdoors in boxes with a 50 cm layer of soil in the experimental field of the Kazan Institute of Biochemistry and Biophysics (Kazan, Russia, 55°47′ N, 49°06′ E) with natural daylight and daily watering. Plants of hops (*Humulus lupulus* L.), hemp (*Cannabis sativa* L.), nettle (*Urtica dioica* L.), sweet clover (*Melilotus officinalis* (L.) Lam.), and wood vetch (*Vicia sylvatica* L.) were collected from the wild. For biochemical analysis, the part of the 65-day-old common bean stem (15–25 cm) from the upper third to the lower third was frozen in liquid nitrogen and stored at −80 °C. Then, the stem was separated by hand into the outer part (phloem with fibers) and the inner part (xylem) (Figure 1a–c). Phloem fiber-rich strips were incubated in boiling 80% ethanol for 10 min to inactivate enzymes capable of degrading polysaccharides. The primary phloem fibers were then separated from the cells of other tissues of the stem cortex (epidermis, collenchyma, sieve tube elements, and parenchyma) by grinding in a mortar with a pestle in 80% ethanol. The number of biological replicates for biochemical analyses was four, each containing five plants.

### 4.2. Light Microscopy and Immunohistochemistry of Common Bean Stem

For light microscopic analysis, the middle part of the upper third of the stem (containing young fibers) and the lower third (containing more mature fibers) of the 65-day-old common bean stem (Figure 1) were fixed in 80% ethanol. For immunohistochemical analysis of the cell walls, the middle part of the non-fixed stem of the common bean was used. Transverse sections (50 µm thick) were prepared with a vibratome Leica VT 1000S (Leica Biosystems, Wetzlar, Germany) using a blade speed of 0.65 mm s^−1^ and a blade frequency of 70 Hz. The stem sections were stained with phloroglucinol-HCl [60] to analyze the presence of lignin. Non-fixed transverse sections for immunohistochemical analysis were incubated for 10 min in 4% (*w*/*v*) paraformaldehyde solution made in 0.2 M phosphate-buffered saline (PBS, pH 7.2).

The immunolabeling procedure was performed using the antibodies INRA-RU2, JIM14, LM2, LM5, LM6, LM11, LM19, LM20, LM21, and LM26 (Table 1). For immunolocalization, sections were blocked with 0.2 M PBS containing 2% (*w*/*v*) bovine serum albumin (BSA) for 30 min at room temperature, incubated with one of the primary monoclonal antibodies diluted 1:5 (JIM14, LM2, LM5, LM6, LM11, LM19, LM20, LM21, and LM26) or 1:3 (INRA-RU2) for 1.5 h at room temperature, then washed three times with PBS and incubated with secondary anti-rat (JIM14, LM2, LM5, LM6, LM11, LM19, LM20, LM21, and LM26) or anti-mouse (INRA-RU2) IgGs conjugated to fluorescein isothiocyanate (FITC; Sigma-Aldrich, St. Louis, MO, USA) diluted 1:100 in PBS for 1 h at room temperature in the dark. Treatment with the primary antibody was omitted for the negative controls. For crystalline cellulose labeling, the carbohydrate-binding module was CBM3a. Sections were blocked with 0.2 M PBS containing 2% (*w*/*v*) bovine serum albumin (BSA) for 30 min at room temperature, incubated with CBM3a at 10 g/mL for 1.5 h at room temperature, then washed three times with PBS and incubated with anti-his mouse antibody (H1029, Sigma Aldrich) diluted 1:1000 for 1 h, then washed three times with PBS and incubated with anti-mouse IgGs conjugated to fluorescein isothiocyanate (FITC; Sigma-Aldrich, St. Louis, MO, USA) diluted 1:100 in PBS for 1 h at room temperature in the dark. After incubation with antibodies, the sections were washed four times in PBS and twice in water. Non-fixed sections were observed using a Leica DM1000 epifluorescence microscope (Leica Biosystems, Wetzlar, Germany) fitted with a mercury lamp and appropriate filter cubes (excitation filter 460–500 nm, barrier filter 512–542 nm).

### 4.3. Confocal Microscopy and Immunohistochemistry of Fibers from Plants Belonging to Malpighiales, Fabales, and Rosales

Segments of the stem middle part of flax, castor bean, sweet clover, hemp, ramie, hops, nettle, storage root of clover, and tendrils of wood vetch were dehydrated in a series of aqueous ethanol solutions (80:20, 60:40, 40:60, 20:80, and 0:100) and gradually impregnated with London Resin White (LRW, EMS, Hatfield, PA, USA) in a series of acetone LRW solutions (80:20, 60:40, 40:60, 20:80, and 0:100). During all these procedures, the samples were stored at +4 °C to avoid resin curing. Samples in 100% resin were kept at room temperature for several hours and then heat cured. Polymerization was performed in flat-bottom Beem capsules (EMS) at 60 °C for 24 h.

Thin sections (700 nm) for immunohistochemistry were prepared using a glass knife on an LKB8800 ultramicrotome (LKB Instruments, Bromma, Sweden) and collected on silane-coated microscope slides (EMSs). Immunolabeling using antibodies LM5, LM26, and INRA-RU2 was performed as described above. Thin sections embedded in resin were observed using an LSM-50 confocal laser scanning microscope (Zeiss, Oberkochen, Germany) with a 488 nm laser and BP 505–530 emission filter (stem cross-sections of flax, castor bean, sweet clover, hemp, ramie, hops, and nettle) or the Leica DM1000 epifluorescence microscope fitted with a mercury lamp and appropriate filter cubes (excitation filter 460–500 nm, barrier filter 512–542 nm) (cross-sections of clover roots and wood vetch tendrils).

### 4.4. In Situ β-Galactosidase Activity

For β-d-galactosidase activity assessment, transverse sections of the stem middle part of flax, hops, and common bean, as well as the storage root of clover and tendrils of wood vetch, were used. Transverse sections (50 µm thick) were prepared with a vibratome Leica VT 1000S (Leica Biosystems, Wetzlar, Germany) using a blade speed of 0.65 mm s^−1^ and a blade frequency of 70 Hz. The resorufin-β-d-galactopyranose (Res-bDGalp) substrate (Merck, Kenilworth, NJ, USA) was resolved in dimethyl sulfoxide and then diluted with 0.2 M PBS (pH 7.2) to 10 mM concentration. Then, 10 µL of the substrate was added to each transverse section. Enzyme activity was detected by red fluorescence on a Leica DM1000 epifluorescence microscope (Leica Biosystems, Wetzlar, Germany) fitted with a mercury lamp and filter cube with excitation at 540–580 nm and extinction at 608–683 nm. Exposure time was maintained constant. The sections were observed after 10 min of reaction. The control sections were heated to 90 °C for 5 min in a water drop and then treated with substrate. The experiment was performed in three biological replicates.

### 4.5. Isolation of Polysaccharide Fractions

Primary phloem fibers of the common bean stem were homogenized in liquid nitrogen with the addition of 10 mM NaOAc buffer (pH 5.0) containing 0.02% NaN_3_. The homogenate was clarified by centrifugation at 8000× *g* for 15 min. The clarified homogenate was then incubated in a boiling water bath for 10 min and mixed with 96% ethanol (final ethanol concentration 80%) at 4 °C overnight to precipitate the buffer-extractable polymers. The sediment was separated by centrifugation at 8000× *g* and 4 °C for 10 min, washed three times with 80% ethanol and once with acetone, and then dried (Figure 4).

Cell wall material from the pellet obtained after homogenization was washed sequentially with water (three times), 80% ethanol, acetone (overnight, 4 °C), water (three times), and 10 mM NaOAc buffer (pH 5.4) with 0.02% NaN_3_ (twice), digested overnight at 45 °C with 0.5% α-amylase from *Aspergillus oryzae* (Megazyme) with 0.02% NaN_3_, washed with the same acetate buffer, water (three times), and acetone, and then dried.

Cell wall polymers were extracted sequentially with 1% ammonium oxalate (boiling water bath, 1 h) and, after washing with water, with 4 M KOH with 3% H_3_BO_4_ (overnight). The KOH fractions were neutralized to pH 7.0 via the addition of acetic acid. The polymers remaining after alkali extraction, which are strongly retained by the cellulose microfibrils, were obtained from the residue by cellulose dissolution, according to the previously described method [11]. Briefly, the cell wall residue was suspended in a solution of 8% LiCl in *N*,*N*-dimethylacetamide for 3 days. Cellulose was precipitated with water and degraded by incubation with 200 µL g^−1^ cellulase from *Trichoderma reesei* (Sigma-Aldrich, Saint Louis, MO, USA) in 10 mM NaOAc buffer (pH 5.4) with 0.02% NaN_3_ at 50 °C for 2 days. Cell wall fractions extracted with ammonium oxalate, alkali, and after cellulose digestion were desalted by passing through a Sephadex G-25M column (8 × 270 mm), concentrated by evaporation at 60 °C, dried, hydrolyzed with 2M TFA at 120 °C for 1 h, and subjected to monosaccharide analysis.

The pellet remaining after cellulose digestion was washed with water, dried, hydrolyzed with 2M TFA, and subjected to monosaccharide analysis to evaluate “lignin-bound polysaccharides”. The difference between the dry mass of the pellet remaining after cellulose digestion and the total yield of monosaccharides obtained after TFA hydrolysis of this fraction was considered as “lignin”. The fractionation procedure is summarized in Figure 4.

The cellulose content was calculated by subtracting the yields of all fractions obtained (buffer-extractable, AO-extractable, KOH-extractable, polysaccharides strongly retained by cellulose, lignin-bound polysaccharides, and lignin) from the dry weight of the initial sample (Table 2).

### 4.6. Size-Exclusion Chromatography

The obtained fractions of buffer-extractable and strongly retained within cell wall polysaccharides were size fractionated in deionized water using an Agilent 1260 Infinity system (Agilent, Santa Clara, CA, USA) equipped with a Sepharose CL-4B column (12 × 400 mm) (Pharmacia, Stockholm, Sweden) at a rate of 0.25 mL/min. The column was calibrated using dextran (2000 kD; Sigma, USA), pullulan standards with molecular masses of 853, 380, 186, 100, and 48 kD (Waters, Milford, MA, USA), and Glc (0.18 kD; Merck, Darmstadt, Germany). To obtain elution profiles after gel filtration, the probes (1 mL) were collected, and the total sugars in each probe were quantified by the phenol-sulfuric acid method [61] using Glc as a standard. The elution profiles presented are the mean values obtained from four independent biological replicates based on the carbohydrate content of the probes and the total amounts of sugars in the fractions.

The probes corresponding to the high-molecular-mass peak in the elution profiles of buffer-soluble and cellulose-retained polymers were combined and concentrated by evaporation at 60 °C. Portions of these preparations were used for monosaccharide determination, immunodot analysis, and structure determination by ^1^H NMR spectroscopy.

### 4.7. Monosaccharide Analysis

Samples (1 mg of dry cell wall material and 40–60 μg of polysaccharide fractions of each type) were hydrolyzed with 2M TFA at 120 °C for 1 h, dried to remove TFA, dissolved in deionized water, and analyzed using high-performance anion-exchange chromatography on an ICS-6000 system (Thermo Fisher Scientific, Waltham, MA, USA) equipped with a CarboPac PA-1 column (4 × 250 mm, Thermo Fisher Scientific, Waltham, MA, USA) with a guard column, using integrated amperometric detection. A quadruple pulse waveform was used for the analysis of carbohydrates by ion chromatography with a gold working electrode. Separation of neutral monosaccharides was performed in 16.5 mM NaOH in isocratic mode over 15 min. The linear gradient of 90% of 15 mM NaOH and 10% of 100 mM NaOH in 1 M NaOAc to 70% of 15 mM NaOH and 30% of 100 mM NaOH in 1 M NaOAc over 10 min was used for the uronic acid determination. The analysis was performed at a flow rate of 1 mL/min and a column temperature of 30 °C. The results were analyzed using the Chromeleon 7.0 software, according to the calibrations obtained for monosaccharide standards pre-treated in advance with 2M TFA at 120 °C for 1 h. Analysis was performed for four independent biological replicates and two analytical replicates. The results of this analysis are presented in tables and diagrams as mean values ± SD.

### 4.8. Immunodot Analysis

Total fractions or subfractions containing 0.5, 1.0, and 2.0 μg of sugar (2 μL) were applied to nitrocellulose membranes (0.2 μm; Sigma). The membranes were air dried for 30 min, washed in PBST (phosphate-buffered saline with 0.05% (*v*/*v*) Triton X-100) for 2 min, blocked with phosphate-buffered saline containing 3% (*w*/*v*) BSA for 1 h, and then incubated with primary monoclonal antibodies for 40 min. The used monoclonal antibodies (Table 1) were JIM14, LM2, LM5, LM6, LM13, LM19, LM20, and LM26, all raised in rats and applied at 1:50 dilution, and INRA-RU2 (mouse, hybridoma supernatant) applied at 1:50 dilution in PBST. After incubation with primary antibodies, the membranes were washed three times for 10 min with PBST and then incubated with corresponding biotinylated secondary antibodies, anti-rat, or anti-mouse (Sigma-Aldrich, Saint Louis, MO, USA) for 40 min. The membranes were washed three times in PBST for 10 min, incubated with streptavidin conjugated to alkaline phosphatase diluted 1:15,000 for 60 min, and developed using a nitroblue tetrazolium/5-bromo-4-chloro-3-indolyl phosphate kit (Sigma-Aldrich, Saint Louis, MO, USA). Potato (*Solanum tuberosum*) galactan and RG-I, wheat (*Triticum aestivum*) flour arabinoxylan, and tamarind (*Tamarindus indica*) seeds XG (all supplied by Megazyme, Bray, Ireland) were used as positive controls.

### 4.9. NMR Spectroscopy

The structure of buffer-extracted high-molecular-weight polymers obtained after the complete destruction of cellulose microfibrils from the cell walls of *Phaseolus vulgaris* stem fibers was characterized by ^1^H NMR spectroscopy. The samples were dissolved in D_2_O (99.9%, Ferak Berlin, Berlin, Germany) to perform the H-D exchange of hydroxyl protons in the polysaccharides, dried, and redissolved in D_2_O (99.994%, Sigma-Aldrich, Saint Louis, MO, USA). NMR spectra were recorded on a Bruker Avance III 600 MHz (Bruker Corporation, Billerica, MA, USA) at 303 K. The residual HOD signal was suppressed by presaturation in all experiments. Data processing and spectra analysis were performed using Topspin 3.6.1 software (Bruker Corporation, Billerica, MA, USA).

### 4.10. Dynamic Light Scattering

The analysis of the hydrodynamic behavior of rhamnogalacturonans I from the fiber cell wall of *Phaseolus vulgaris* was determined by dynamic light scattering. The experiments were carried out on a Photocor Complex spectrometer (Photocor Instruments Inc., Moscow, Russia) equipped with a compact goniometer, a real-time correlator (200 channels; fastest sampling period 10 ns), a thermostat, and a monochromatic laser light operating at 657.29 nm. All measurements were performed in deionized water (type A, H_2_O-MA-UV-T system, Sartorius, Germany) at 20 °C and a scattering angle of 140°. Polysaccharide concentrations ranged from 0.1 to 6.2 mg/mL for buffer-extractable RG-I and from 0.1 to 1.6 mg/mL for RG-I released after complete destruction of cellulose. Prior to analysis, the solvent and samples were filtered through a 0.22 µm polytetrafluoroethylene (PTFE) membrane. Autocorrelation functions were recorded during an accumulation time of 40–140 s using Photocor-FC 7.0 software (Photocor Instruments Inc., Moscow, Russia). Each autocorrelation function was averaged from 10–20 measurements. The z-averaged hydrodynamic radius (R_h_) was calculated from the Stokes–Einstein relation. The standard values for viscosity and refractive index of water at 20 °C were used to calculate particle sizes. The data were processed by the multi-pass distribution analysis algorithm using the DynaLS software package, version 2.8.3 (Dr. Alexander Goldin (Alano Ltd., Hefa, Israil).

## 5. Conclusions

Tertiary cell wall deposited in fibers of many plant species is a spectacular example of special cell wall design to meet a specific function. The obtained results confirm that RG-I with β-(1,4)-galactan side chains is the must-have component of this highly specialized cell wall present in fibers of many plant species. The molecules of RG-I isolated from fibers with tertiary cell wall are able to form strong molecular aggregates. The details of the RG-I structure may vary by the proportion of homogalacturonan part in the backbone of the polymer, by the degree of RG-I branching and by the presence of arabinose in the side chains. These parameters can be related to the properties of fibers, both in planta to provide contractile forces and in isolated technical fibers used for application purposes.

The obtained results also help to find the adequate criteria to identify the tertiary cell wall, which are still debated [1,3,4]. We consider the presence of RG-I in the thickened inner layer of the fiber cell wall among the most reliable criteria. The easiest approach to revealing such polysaccharides in the thickened cell wall is staining with dyes, such as toluidine blue, which reveals acidic polymers [1,16]. The stricter approach is immunolabeling using RG-I-related antibodies, like INRA-RU2, LM5, and LM26. However, it is necessary to use at least two of these antibodies, since the labeling of RG-I backbones or side chains on tissue sections can be limited, as illustrated by the data in the current paper.

## Figures and Tables

**Table 1:** Antibodies and CBM used for immunohistochemistry and immunoblot analyses.

Antibody/CBM	Specificity	References
INRA-RU2 ^1^	Rhamnogalacturonan I backbone	[27]
LM5 ^2^	β-(1,4)-d-Galactan	[28]
LM26 ^2^	β-(1,6)-Galactosyl substitution of β-(1,4)-d-galactan	[29]
LM6 ^2^	Prefer α-(1,5)-l-arabinan, may bind to arabinogalactan protein	[30,31]
LM13 ^2^	α-(1,5)-l-Arabinan (linear)	[31,32]
LM2 ^2^	Arabinogalactan protein	[33,34]
JIM14 ^2^	Arabinogalactan, arabinogalactan protein	[34,35,36]
LM19 ^2^	Low-esterified homogalacturonan	[37]
LM20 ^2^	High-esterified homogalacturonan	[37]
LM11 ^2^	Xylan, arabinoxylan	[38]
LM21 ^2^	Heteromannan	[39]
CBM3a ^2^	Planar surface of crystalline cellulose	[40]

^1^ Kindly provided by Dr. Fabienne Guillon, INRA, Nantes, France; ^2^ Kindly provided by Prof. Paul Knox, University of Leeds, UK.

**Table 2:** Yields of cell wall fractions and monosaccharide composition of TFA-hydrolysable polysaccharides from the total cell wall of common bean fibers.

Cell Wall Fractions, % of Dry Weight of Cell Wall ^1^						
Buffer-Extractable Polysaccharides ^2^	AO-Extractable Polysaccharides ^2^	KOH-Extractable Polysaccharides ^2^	Poly-Saccharides Strongly Retained by Cellulose (without Glc) ^2^	Lignin-Bound Poly-Saccharides ^3^	Cellulose ^4^	Lignin ^5^
4.2 ± 1.9	4.7 ± 1.8	6.0 ± 1.2	3.7 ± 0.7	0.3 ± 0.1	74.7 ± 2.8	6.4 ± 0.9
**Cell wall monosaccharide proportion, mol% ^1^**						
**Rha**	**Ara**	**Gal**	**Glc**	**Xyl + Man**	**GalA**	**GlcA**
4.2 ± 0.1	6.6 ± 1.4	4.3 ± 1.7	63.3 ± 7.7	11.2 ± 2.7	9.9 ± 5.7	0.5 ± 0.1

^1^ Data are presented as mean values ± SD. ^2^ Determined as the sum of the yields of all monosaccharides obtained by TFA hydrolysis from the corresponding fraction. ^3^ Determined as the sum of the yields of all monosaccharides obtained by TFA hydrolysis from the pellet remaining after cellulose digestion. ^4^ Calculated from the dry weight of the initial sample by subtracting yields of buffer-extractable, AO-extractable, KOH-extractable, polysaccharides strongly retained by cellulose, lignin-bound polysaccharides, and lignin. ^5^ Calculated from the dry mass of the pellet remaining after cellulose digestion by subtracting the yields of monosaccharide obtained by TFA hydrolysis from this pellet. Rha—rhamnose; Ara—arabinose, Gal—galactose, Glc—glucose, Xyl + Man—xylose and mannose, GalA—galacturonic acid, GlcA—glucuronic acid.

## Data Availability

The data presented in this study are available on request from the corresponding author.
